# Strict glycemic control to prevent surgical site infections in gastroenterological surgery

**DOI:** 10.1002/ags3.12006

**Published:** 2017-04-25

**Authors:** Yoshio Takesue, Toshie Tsuchida

**Affiliations:** ^1^ Department of Infection Prevention and Control Hyogo College of Medicine Hyogo Japan; ^2^ Department of Nursing Hyogo University of Health Sciences Hyogo Japan

**Keywords:** diabetes, gastroenterological surgery, hyperglycemia, surgical site infection, tight glycemic control

## Abstract

Perioperative hyperglycemia is a risk factor for surgical site infections (SSI). Although the recommended target blood glucose level (BG) is 140–180 mg/dL for critically ill patients, recent studies conducted in patients undergoing surgery showed a significant benefit of intensive insulin therapy for the management of perioperative hyperglycemia. The aim of the present review is to evaluate the benefits of strict glycemic control for reducing SSI in gastroenterological surgery. We carried out a post‐hoc analysis of the previously published data from research on the risk factors for SSI. The highest BG within 24 hours after surgery was evaluated. A total of 1555 patients were enrolled in the study. In multivariate analysis, a dose–response relationship between the level of hyperglycemia and the odds of SSI was demonstrated when compared with the reference group (≤150 mg/dL) (odds ratio [OR] = 1.68, 95% confidence interval [CI] 1.14–2.49 for 150–200 mg/dL; and OR = 2.15, 95% CI 1.40–3.29 for >200 mg/dL). Unexpectedly, hyperglycemia was not a significant risk factor for SSI among diabetes patients. By contrast, non‐diabetes patients with a BG of >150 mg/dL were found to have increased odds of SSI. In conclusion, a target BG of ≤150 mg/dL is recommended in patients without diabetes who undergo gastroenterological surgery. Additional study is required to determine an optimal target BG in diabetes patients. Because of the risk of hypoglycemia, a conventional protocol is indicated for patients admitted to the general ward where frequent glucose measurement is not assured.

## 1 Introduction

Increasing glucose concentrations exert multiple substantial and opposing effects on several well‐recognized cellular and immunological parameters.^1^ Most importantly, acute, short‐term hyperglycemia affects all major components of innate immunity and impairs the ability of the host to combat infection. In surgical patients, release of counter‐regulatory catabolic hormones leads to hyperglycemia. In addition, the stress response to surgical insult results in insulin tolerance, and decreased pancreatic beta‐cell function causes hypo‐insulinemia, augmenting stress‐induced hyperglycemia.^2^ For the prevention of surgical site infections (SSI), appropriate perioperative insulin therapy is required in patients with hyperglycemia.^3^, ^4^, ^5^, ^6^, ^7^

Data regarding the impact of long‐term glucose control on SSI have been conflicting in patients with diabetes mellitus (DM). In DM patients who underwent major non‐cardiac surgery, a hemoglobin (Hb) A1c level of <7% was significantly associated with decreased infectious complications with an adjusted odds ratio (OR) of 2.13.^9^ In contrast, Latham *et al*.^8^ reported that DM and postoperative hyperglycemia were independently associated with the development of SSI. However, among DM patients, elevated Hb Alc values were not associated with a statistically significantly increased risk of infection. Acott *et al*.^10^ also described that there was no correlation between risk of complication and Hb A1c level. These reports suggest that short‐term perioperative glucose control may be more impactful in decreasing SSI than long‐term control of Hb A1c.

Earlier guidelines for prevention of SSI from the Centers for Disease Prevention and Control (CDC)^3^ published in 1999 recommended perioperative treatment of hyperglycemia (≥200 mg/dL) in patients with DM. The Surgical Care Improvement Project^5^ (SCIP) developed a quality performance measure to maintain the 6 a.m. controlled blood glucose level (BG) at <200 mg/dL in cardiac surgery (<180 mg/dL in the updated version^11^). This recommendation has been challenged by recent studies suggesting that an even lower target BG is required to prevent SSI.^12^, ^13^, ^14^, ^15^, ^16^, ^17^, ^18^ The intensive insulin administration protocol (intensive protocol), however, leads to an increased risk of hypoglycemia, which, in turn, is associated with increased morbidity and mortality.^19^, ^20^ It seems that very strict glycemic control has modest benefits, with adverse effects often outweighing these advantages in critically ill patients. However, recent studies have indicated differing results for certain patient subgroups, such as surgical patients with acute operative hyperglycemia in the immediate postoperative period.^12^, ^13^, ^14^, ^15^, ^16^

As patients with DM have a higher risk of cardiovascular disease, and the association with SSI was significantly higher for cardiac surgery compared with other surgeries in DM patients^21^ (OR 2.03, 95% confidence interval [CI] 1.13–4.05), many of the available studies evaluating the efficacy of glycemic control on SSI were limited to cardiac surgery patients,^22^ which raised questions about the generalizability of the results to patients undergoing other surgical procedures.^12^ Vigorous studies have been recently conducted to clarify the efficacy of tight (strict) glycemic control in patients undergoing gastroenterological surgery, and four of 15 randomized clinical trials (RCT) comparing intensive with conventional protocols were carried out in patients undergoing abdominal surgery (nine were conducted for cardiac surgery).^23^ The definition of intensive protocol varies from ‘moderately strict glycemic control’ with an upper limit target of 150 mg/dL to ‘very strict control’ with a target of 110 mg/dL (Table 1). The aim of the present review is to evaluate the benefits of the intensive protocol for reducing SSI in gastroenterological surgery. This review also aims to clarify the impact of hyperglycemia on SSI according to the DM status.

**Table 1:** Definition of insulin therapy and glycemic control

Insulin therapy	Glycemic control	Upper limit target blood glucose level (mg/dL)
Intensive protocols	Moderately strict	150
	Very strict	110
Conventional protocols	High concentration	200
	Moderate concentration	180

## 2 Indication for intensive protocol in critically ill patients and in those undergoing surgery

### 2.1 Critically ill patients

An earlier RCT^24^ has shown that the intensive protocol targeted to very strict control (80–110 mg/dL) reduced mortality in critically ill patients in the surgical intensive care unit (ICU), compared with the conventional protocol with high BG control (180–200 mg/dL). However, several subsequent studies including an RCT conducted in the medical ICU have failed to demonstrate reduced mortality in a group under very strict control.^25^, ^26^, ^27^ The NICE‐SUGAR trial^19^ comparing a very strict protocol and a conventional protocol with moderate BG control (<180 mg/dL) demonstrated a substantially increased mortality rate in the study group. In recent years, several meta‐analyses have confirmed detrimental effects for very strict control when using mortality as an endpoint, and have shown the risk of hypoglycemia affecting the mortality (Table 2.)^26^, ^28^, ^29^

**Table 2:** Clinical outcomes of intensive insulin therapy and recommendation for glycemic control in patients undergoing surgery and in those who are critically ill

Subject	Primary endpoint	Blood glucose level and clinical outcomes	Duration of glycemic control	Potential consequence of hypoglycemic events by intensive protocol	Recommendation for glycemic control	
					Terms	Target range of blood glucose level (mg/dL)
Patients undergoing surgery	Postoperative complications, SSI	Dose–response relationship between blood glucose levels and SSI	Immediate postoperative period	Although a significantly higher risk of hypoglycemia was found, the protocol did not increase mortality	Intensive protocol targeted to moderately strict glycemic control	110–150
Critically ill patients including those with sepsis	Mortality	Target of <180 mg/dL showed lower mortality than that of 81–108 mg/dL	During ICU stay	Hypoglycemia was an independent factor for mortality	Conventional protocol targeted to moderate concentration glycemic control	140–180

ICU, intensive care unit; SSI, surgical site infection.

The NICE‐SUGAR trial,^19^ which included a relatively small number of patients with elective surgery (12.5%), was carried out in critically ill patients at high risk of death (Acute Physiology and Chronic Health Evaluation [APACHE] II score, 21.1; death at day 28, 21.5%), and 21.6% of the patients already had sepsis at the time of randomization. A post‐hoc analysis of the NICE‐SUGAR study database^20^ showed that very strict control leads to moderate and severe hypoglycemia, both of which are associated with an increased risk of death, whereas high incidence of hypoglycemia possibly leads to increased mortality in patients assigned to a group under very strict control.

Therefore, it would be prudent to ensure that strategies for managing the BG in critically ill patients focus not only on the control of hyperglycemia but also on avoidance of hypoglycemia. The clustered ranking plot reported by Yamada *et al*.^28^ provided precise risk estimates and better information about the hierarchy of target BG ranges for achieving safe and effective glycemic control in critically ill patients, and a BG of 140–180 mg/dL achieved the best outcome in relation to all‐cause mortality and hypoglycemia. Surviving Sepsis Campaign guidelines^30^ for the management of severe sepsis and septic shock recommended a protocolized approach with a target upper BG of 180 mg/dL rather than 110 mg/dL.

### 2.2 Patients undergoing surgery

There are currently several debates regarding the benefits of strict glucose control in less critically ill patients undergoing elective surgery as opposed to critically ill (medical) patients^12^, ^13^, ^14^, ^15^, ^16^, ^17^, ^18^ (Table 2). Although the recommended target BG is 140–180 mg/dL for most ICU patients,^27^ a recent meta‐analysis^23^ comparing the efficacy between an intensive and a conventional protocol in patients undergoing surgery showed a significant benefit for the intensive protocol in reducing SSI. Beneficial effects in reducing SSI were confirmed in studies with very strict and moderately strict control, and the effect was similar in both groups (*P* = 0.328).^6^

Although a higher risk of hypoglycemic events was observed with the intensive protocol, the protocol including a very strict control group did not increase the risk of postoperative death and stroke compared with the conventional protocol in surgical patients,^23^ and it was concluded that an intensive protocol can be carried out safely without the risk of serious adverse events in surgical patients (Table 2). The result of this meta‐analysis, however, should be interpreted with caution. Most of the studies included were done in patients undergoing cardiac surgery or major gastrointestinal surgeries with a substantial proportion of the study population having a postoperative ICU stay. In the ICU, a high adherence rate to the insulin treatment regimen and BG measurement protocol is assured. It remains unknown whether the results can be extrapolated to a more general population.

In global guidelines for the prevention of SSI by the World Health Organization (WHO),^6^ the panel suggested the use of perioperative intensive protocols for patients undergoing surgical procedures to reduce the risk of SSI. The CDC members of the WHO guidelines panel decided that the available evidence did not allow the definition of an optimal target BG, and emphasized that hypoglycemia associated with intensive protocols carries a serious risk of life‐threatening complications.^6^ By contrast, the guidelines by the American College of Surgeons (ACS) and Surgical Infection Society (SIS)^7^ specified the optimal target BG, and recommended that perioperative glycemic control should be between 110 and 150 mg/dL (moderately strict control) except in cardiac surgery patients for whom the target is <180 mg/dL, because a target BG of <110 mg/dL has been linked to adverse outcomes and increased episodes of hypoglycemia and do not decrease SSI risk compared with moderately strict control.

## 3 Relationship between perioperative BG and SSI in patients who undergo gastroenterological surgery

We previously reported independent risk factors associated with SSI in patients who underwent gastroenterological surgery^31^ (approved by the research ethics committees of Hyogo College of Medicine, No. 1088). To better understand the results, we carried out a post‐hoc analysis of the data to explore the relationship between postoperative BG and SSI in this article (No. 2572). A total of 1555 patients who underwent esophagectomy (75), gastrectomy (360), colon and rectal surgery (745), and hepatobiliary‐pancreatic surgery (375) in Hyogo College of Medicine were enrolled in the study. Patients with laparoscopic cholecystectomy and those who died within 4 days after surgery were excluded. The highest BG within 24 h after surgery were evaluated. The diagnosis of SSI was made based on definitions stated in the guidelines issued by the National Healthcare Safety Network.^32^

BG >200 mg/dL, which has been traditionally defined as clinically significant hyperglycemia, was observed in 28.1% of patients. SSI occurred in 263 of 1555 patients (16.9%) and 119 of them had incisional SSI, whereas 180 had organ/space SSI (36 had both). Independent factors associated with BG >200 mg/dL are shown in Table 3. Among surgical procedures, hepatobiliary‐pancreatic surgery and esophagectomy were independent risk factors for postoperative hyperglycemia.

**Table 3:** Independent factors associated with postoperative hyperglycemia (>200 mg/dL) in patients undergoing gastroenterological surgery

Factors	Odds ratio	95% CI	*P*‐value
Age >65 years	1.80	1.38–2.36	<0.001
Body mass index >25	2.83	1.31–6.09	0.008
Hypertension	1.40	1.07–1.84	0.014
Diabetes	8.22	5.51–12.28	<0.001
Preoperative anemia	1.38	1.07–1.78	0.013
Esophagectomy	9.02	5.32–15.28	<0.001
Hepatobiliary‐pancreatic surgery	2.11	1.56–2.88	<0.001
Prolonged surgery	1.33	1.00–1.78	0.053
Laparoscopic surgery	0.35	0.18–0.65	0.001

95% CI, 95% confidence interval.

### 3.1 Optimal perioperative target BG to prevent SSI in patients undergoing gastroenterological surgery

**Figure 1:**
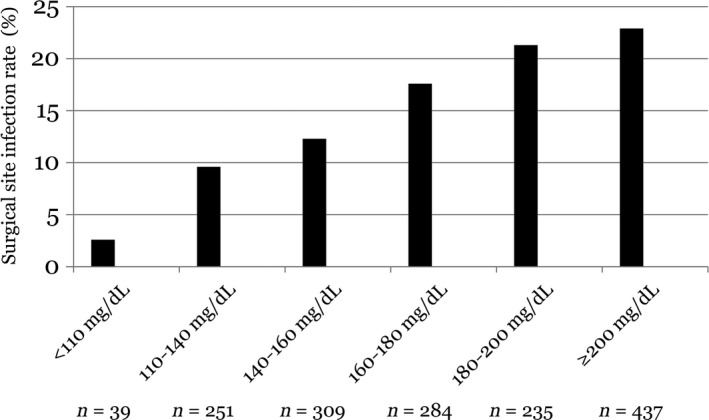
Surgical site infection rate according to the postoperative blood glucose concentration in patients who underwent gastroenterological surgery

SSI rates increased incrementally for patients with higher BG categories (Figure 1). The incidence of SSI ranged from 2.6% in the ≤110 mg/dL category to 22.9% in the >200 mg/dL category, and the dose–response relationship was confirmed by the residual analysis (Table 4). In multivariate analysis, a dose–response relationship between the level of hyperglycemia and the odds of SSI was also demonstrated when compared with the reference group (≤150 mg/dL) (OR = 1.68 [95% CI 1.14–2.49] for 150–200 mg/dL; and OR = 2.15 [95% CI 1.40–3.29] for >200 mg/dL) (Table 5). The cut‐off target BG for SSI was evaluated using the receiver‐operating characteristic (ROC) curve. Area under the ROC curve was 0.615. Although the accuracy was poor, the cut‐off value was considered to be 150 mg/dL from real data showing sensitivity and specificity at various cut‐off points of BG. In multivariate analysis, BG level of >150 mg/dL was a significant predictor of SSI (OR 1.81, 95% CI 1.26–2.60) (Table 6). If target BG ≤150 mg/dL is adopted, 70.5% of patients are candidates for postoperative glycemic control in gastroenterological surgery.

**Table 4:** Surgical site infection rate according to postoperative blood glucose concentration in patients who underwent gastroenterological surgery

Postoperative blood glucose level (mg/dL)	SSI rate	Adjusted residuals
>200	100/437 (22.9%)	3.2
181–200	50/235 (21.3%)	2.1
161–180	50/284 (17.6%)	0.5
141–160	38/309 (12.3%)	–2.2
111–140	24/251 (9.6%)	–3.2
≤110	1/39 (2.6%)	–2.4

Significant level of residuals: ± 1.96, *P* < 0.05; ± 2.58, *P* < 0.01.

**Table 5:** Multivariate analysis on the effect of each blood glucose concentration category on surgical site infections in patients with and without diabetes who underwent gastroenterological surgery

Subjects	Odds ratio for SSI in each blood glucose level category (95% CI, *P*‐value)		
	≤150 (*n* = 458)	151–200 (*n* = 669)	>200 (*n* = 428)
Diabetes patients (*n* = 155)	Reference	2.35 (0.03–171.51, 0.696)	1.50 (0.16–14.09, 0.723)
Non‐diabetes patients (*n* = 1400)	Reference	1.73 (1.16–2.57, 0.007)	1.74 (1.08–2.81, 0.024)
Overall (*n* = 1555)	Reference	1.68 (1.14–2.49, 0.009)	2.15 (1.40–3.29, <0.001)

95% CI, 95% confidence interval.

**Table 6:** Multivariate analysis of independent factors associated with surgical site infection

Factor	Odds ratio	95% CI	*P*‐value
Male	1.53	1.13–2.08	0.006
Chronic liver dysfunction	1.49	1.05–2.11	0.025
Postoperative blood glucose level >150 mg/dL	1.81	1.26–2.60	0.001
Wound class 3, 4	2.50	1.23–5.08	0.011
Esophagectomy	6.40	3.74–11.00	<0.001
Stoma construction	2.76	1.96–3.88	<0.001
Prolonged surgery	2.01	1.48–2.73	<0.001
Laparoscopic surgery	0.25	0.10–0.66	0.005

95% CI, 95% confidence interval.

Ata *et al*.^14^ reported that a glucose level >140 mg/dL was the only significant risk factor associated with SSI in patients undergoing colorectal surgery. Meta‐analysis^23^ confirmed the benefits of an intensive protocol targeting <150 mg/dL for reducing SSI, and recent guidelines^7^ recommended that perioperative glycemic control should be between 110 and 150 mg/dL. A dose–response relationship between BG level and SSI rate was reported in general surgery patients.^14^ Bivariate analysis revealed that compared with patients with a first postoperative BG ≤110 mg/dL, the likelihood of acquiring an SSI increased progressively for patients with higher BG (OR 3.61 for 111–140 mg/dL; OR 6.26 for 141–180 mg/dL; OR 5.92 for 181–220 mg/dL; and OR 12.13 for >220 mg/dL). In a logistic model, Ramos *et al*.^15^ reported that every 40 mg/dL increase from normoglycemia (<110 mg/dL) led to a 30% increased risk of infection. Kwon *et al*.^13^ also found a clear dose–response relationship between BG and SSI in favor of a lower BG level. Interestingly, Okabayashi *et al*.^33^ examined the efficacy of very strict perioperative control targeting 80–110 mg/dL using an artificial pancreas with a closed‐loop glycemic control system in patients undergoing hepatobiliary‐pancreatic surgery, which resulted in a significantly lower rate of SSI and pancreatic fistula compared with a target of 140–180 mg/dL.

### 3.2 Association of perioperative hyperglycemia with risk of SSI among patients with and without DM

In our study, 155 of 1555 patients (10.0%) had DM. The SSI rate was significantly higher in DM patients than in non‐DM patients (22.6% *vs* 16.2%; *P *= 0.047). The incidence of postoperative BG >200 mg/dL (>150 mg/dL) was 73.5% (94.2%) in DM patients and 22.4% (67.9%) in non‐DM patients. Postoperative hyperglycemia occurred in a considerable number of non‐DM patients in addition to known DM patients undergoing gastroenterological surgery. Unexpectedly, hyperglycemia was not a significant risk factor for SSI among DM patients (OR = 1.48 [95% CI 0.13–16.74, *P* = 0.752] for >150 mg/dL; OR = 1.74 [95% CI, 0.65–4.67, *P *= 0.275] for >200 mg/dL). By contrast, non‐DM patients with a BG >150 mg/dL were found to have increased odds of SSI. However, a BG >200 mg/dL was not selected as a significant risk factor for SSI (OR = 1.75 [95% CI 1.20–2.54, *P *= 0.004] for >150 mg/dL; OR = 1.32 [95% CI 0.94–1.86, *P *=* *0.111] for >200 mg/dL).

Patients were classified into three categories according to the BG level (≤150 mg/dL [reference]; 151–200 mg/dL; and >200 mg/dL), and the effect of hyperglycemia on the odds of SSI was analyzed (Table 5). Patients in both of the higher BG categories had significantly increased odds of SSI among non‐DM patients. However, increased odds of SSI in a dose–response manner for each increasing level of hyperglycemia category in the overall patient analysis was not demonstrated in non‐DM patients.

Systematic review and meta‐analysis addressed a significant association between DM and SSI,^21^ and hyperglycemia in DM patients who undergo surgery is associated with increased rates of SSI. Although non‐DM patients had a lower incidence and decreased severity of hyperglycemia compared with DM patients, hyperglycemia was also associated with adverse outcomes in non‐DM patients.^13^, ^16^, ^17^ In the assessment of operative‐day BG in non‐DM patients who underwent colectomy, normoglycemia (≤120 mg/dL) endured in 26.2%, whereas 53.6% had BG of 121–160 mg/dL, 15.1% had 161–200 mg/dL, and 4.5% >200 mg/dL.^18^ Similarly, Kiran *et al*.^16^ demonstrated that 66.7% of non‐DM patients who underwent colorectal surgery experienced hyperglycemia (>125 mg/dL) which was independently associated with septic complications. A meta‐analysis by de Vries *et al*.^23^ showed that the benefit of an intensive protocol over a conventional protocol in reducing SSI was consistent in patients both with and without DM.

In contrast, Kotagal *et al*.^17^ reported that the risk of complications was linked to hyperglycemia for non‐DM patients but not for DM patients, which is consistent with our results. The risk of complications increased in a dose–response manner at each level of hyperglycemia in non‐DM patients, with an OR of 1.26 for 125–180 mg/dL, and 1.63 for >180 mg/dL when compared with the reference group (≤125 mg/dL). Kwon *et al*.^13^ demonstrated that among patients with hyperglycemia, non‐DM patients had a worse outcome than‐DM patients in general surgery. Frisch *et al*.^12^ found that the risk of death increased in proportion to perioperative BG for non‐DM patients only, and the association between increased risk of complications and a BG of >150 mg/dL existed particularly in non‐DM patients. Interestingly, van den Berghe *et al*.^34^ mentioned that an intensive protocol reduces the mortality of all medical/surgical ICU patients except those with a prior history of DM.

There are three possible theories for this diabetes paradox:^14^ (i) a higher level of surgical insult that causes a non‐DM patient to have the same level of hyperglycemia as a DM patient; (ii) underuse of insulin in non‐DM patients; and (iii) the possibility that DM patients have an adaptation to hyperglycemia. A single initial or maximal postoperative elevated BG was evaluated in several studies including by us, and corrected BG by insulin therapy was not taken into account. Kotagal *et al*.^17^ reported that DM patients were significantly more likely to receive insulin than non‐DM patients at each level of hyperglycemia. With adequate glycemic control by the use of insulin, initial BG level is considered not to have a significant effect on SSI in DM patients.

If the second theory is true, appropriate use of insulin for non‐DM patients is a target for quality improvement. In fact, Kwon *et al*.^13^ found that those with hyperglycemia on the day of surgery who received insulin had no significant increase in infection. Furthermore, among patients who had hyperglycemia and received insulin, those whose BG was corrected had significantly lower rates of adverse events than those who had persistent hyperglycemia in both DM and non‐DM populations.^17^ These findings strongly support the monitoring of BG level and early consideration of management strategies for glycemic control after surgery, even in non‐DM patients. Guidelines by WHO^6^ and ACS/SIS^7^ suggest an intensive protocol for both DM and non‐DM adult patients undergoing surgical procedures to reduce the risk of SSI.

## 4 Insulin therapy in patients with perioperative hyperglycemia

When insulin was given to surgical patients with hyperglycemia, which was otherwise an independent factor for several adverse events including infections, the odds for infection were no longer significant.^10^ Important processes of care for insulin therapy include use of a validated insulin titration program, frequent BG monitoring, and avoidance of finger‐stick glucose testing through the use of arterial or venous glucose samples.^35^ Furnary *et al*.^36^ reported that glucose control was significantly better with continuous insulin infusion than with s.c. insulin, and multivariable analysis showed that continuous insulin infusion was independently protective against death. Among 15 RCT comparing intensive and conventional protocols, the intensive group in all studies used i.v. insulin administration (continuous administration in nine studies), whereas three studies used s.c. administration in the conventional group.^23^

Table 7 shows the glucose management protocol by continuous insulin infusion to achieve the target BG of ≤150 mg/dL in surgical patients admitted to the ICU.^37^ Patients on insulin infusions must have their BG monitored every 1–2 h until BG level and insulin infusion rates are stable, and then every 4 h thereafter. BG level obtained with point‐of‐care testing of capillary blood may not accurately estimate plasma glucose values. BG are measured with the use of arterial blood gas analyzers or laboratory analyzers whenever possible. The standard dilution of insulin is 50 units of regular insulin in 50 mL of normal saline (1 unit/mL). Insulin infusions should be titrated using the graded series of algorithms (Table 7).

**Table 7:** Glucose management protocol to achieve target glucose level of ≤150 mg/dL in surgical patients admitted to the ICU

Blood glucose level (mg/dL)	Monitoring of blood glucose level	Insulin infusion rate (units/h)			
		Algorithm 1	Algorithm 2	Algorithm 3	Algorithm 4
≤60	Treat as hypoglycemia				
61–90	Every 1 h	Off	Off	Off	Off
91–110	Every 2 h	Off	0.5^a^	1^a^	1.5^a^
111–120	Every 2 h	Off	1^a^	2^a^	3^a^
121–150	Every 2 h	Off	1^a^	3^a^	5^a^
151–180	Every 2 h	1	2	4	7
181–210	Every 2 h	2	3	5	9
211–240	Every 2 h	2	4	6	12
241–270	Every 2 h	3	5	8	16
271–300	Every 1 h	3	6	10	20
301–330	Every 1 h	4	7	12	24
331–360	Every 1 h	4	8	14	24
>360	Every 1 h	6	12	16	28

Algorithm 1: All patients to start on this algorithm excluding diabetes patients previously on insulin. Algorithm 2: Patients already on insulin prior to ICU admission, or patients not controlled on algorithm 1. Algorithm 3: Patients not controlled on algorithm 2. Algorithm 4: Patients not controlled on algorithm 3.

1. Move up by one algorithm if glucose levels are >180 mg/dL on two consecutive measurements after titrating insulin.

2. Move down by one algorithm if glucose levels drop below 90 mg/dL on two consecutive measurements or decrease by more than 100 mg/dL in 1 h.

a Diabetes patients previously on insulin.

Patients admitted with diabetic coma are excluded from this protocol.

ICU, intensive care unit.

Recently, a computer‐based insulin infusion protocol has been introduced in the ICU setting, for which improved glycemic control over the manual protocol was reported.^38^ Saur *et al*.^39^ described that surgical ICU patients who were managed using a software‐guided program achieved tighter glycemic control and fewer glycemic derangements than those managed with the paper‐based insulin dosing regimen. In computer‐based systems, the initiation screen requires entry of the current BG as well as target high and low BG limits. After verifying protocol parameters the physician clicks ‘calculate drip rate’ and the insulin drip rate is printed on the screen. With subsequent bedside BG testing, nurses enter protocol‐mandated glucose reading into the system's ‘titration screen’, and adjust the insulin drip rates based on the recommendations provided.^38^

## 5 Conclusions

A target BG of ≤150 mg/dL is recommended in non‐DM patients who undergo gastroenterological surgery. Additional study is required to determine an optimal target BG for surgical DM patients. In contrast, a target BG level of 140–180 mg/dL is recommended in critically ill patients such as those with postoperative complications including infections. Because of the risk of hypoglycemia, a conventional protocol is indicated for patients admitted to the general ward where frequent glucose measurement is not assured. To achieve the target BG and minimize the risk of hypoglycemia in the intensive protocol, it is necessary to improve current practice in surgical patients by ensuring the appropriate insulin delivery and management protocol according to the best available evidence. Further RCT comparing moderately strict control and very strict control are needed to clarify the optimal intensive protocol that reveals a beneficial effect in reducing SSI without a significantly increased risk of hypoglycemia.

## Conflicts of Interest

Authors declare no conflicts of interest for this article.

## References

[1] Turina M , Fry DE , Polk HC Jr . Acute hyperglycemia and the innate immune system: clinical, cellular, and molecular aspects. Crit Care Med 2005;33:1624–33. 16003073 10.1097/01.ccm.0000170106.61978.d8

[2] Qadan M , Weller EB , Gardner SA , Maldonado C , Fry DE , Polk HC Jr . Glucose and surgical sepsis: a study of underlying immunologic mechanisms. J Am Coll Surg 2010;210:966–74. 20510806 10.1016/j.jamcollsurg.2010.02.001

[3] Mangram AJ , Horan TC , Pearson ML , Silver LC , Jarvis WR . Guideline for prevention of surgical site infection, 1999. Centers for Disease Control and Prevention (CDC) Hospital Infection Control Practices Advisory Committee. Am J Infect Control 1999;27:97–132. 10196487

[4] Anderson DJ , Podgorny K , Berríos‐Torres SI , et al. Strategies to prevent surgical site infections in acute care hospitals: 2014 update. Infect Control Hosp Epidemiol 2014;35:605–27. 24799638 10.1086/676022 PMC4267723

[5] Bratzler DW . Surgical care improvement project performance measures: good but not perfect. Clin Infect Dis 2013;56:428–9. 23143098 10.1093/cid/cis944

[6] World Health Organization . Accessed December 19, 2016. Available from http://apps.who.int/iris/bitstream/10665/250680/1/9789241549882-eng.pdf?ua=1.

[7] Ban KA , Minei JP , Laronga C , et al. American College of Surgeons and Surgical Infection Society: Surgical site infection guidelines, 2016 Update. J Am Coll Surg 2017;224:59–74. 27915053 10.1016/j.jamcollsurg.2016.10.029

[8] Dronge AS , Perkal MF , Kancir S , Concato J , Aslan M , Rosenthal RA . Long‐term glycemic control and postoperative infectious complications. Arch Surg 2006;141:375–80. 16618895 10.1001/archsurg.141.4.375

[9] Latham R , Lancaster AD , Covington JF , Pirolo JS , Thomas CS Jr . The association of diabetes and glucose control with surgical‐site infections among cardiothoracic surgery patients. Infect Control Hosp Epidemiol 2001;22:607–12. 11776345 10.1086/501830

[10] Acott AA , Theus SA , Kim LT . Long‐term glucose control and risk of perioperative complications. Am J Surg 2009;198:596–9. 19887184 10.1016/j.amjsurg.2009.07.015

[11] QualityNet. Surgical Care Improvement Project . Accessed January 15, 2017. Available from http://www.qualitynet.org/dcs/ContentServer?c=Page&pagename=QnetPublic%2FPage%2FQnetTier3&cid=122877356487.

[12] Frisch A , Chandra P , Smiley D , et al. Prevalence and clinical outcome of hyperglycemia in the perioperative period in noncardiac surgery. Diabetes Care 2010;33:1783–8. 20435798 10.2337/dc10-0304 PMC2909062

[13] Kwon S , Thompson R , Dellinger P , Yanez D , Farrohki E , Flum D . Importance of perioperative glycemic control in general surgery: a report from the Surgical Care and Outcomes Assessment Program. Ann Surg 2013;257:8–14. 23235393 10.1097/SLA.0b013e31827b6bbc PMC4208433

[14] Ata A , Lee J , Bestle SL , Desemone J , Stain SC . Postoperative hyperglycemia and surgical site infection in general surgery patients. Arch Surg 2010;145:858–64. 20855756 10.1001/archsurg.2010.179

[15] Ramos M , Khalpey Z , Lipsitz S , et al. Relationship of perioperative hyperglycemia and postoperative infections in patients who undergo general and vascular surgery. Ann Surg 2008;248:585–91. 18936571 10.1097/SLA.0b013e31818990d1

[16] Kiran RP , Turina M , Hammel J , Fazio V . The clinical significance of an elevated postoperative glucose value in nondiabetic patients after colorectal surgery: evidence for the need for tight glucose control? Ann Surg 2013;258:599–604. 23979274 10.1097/SLA.0b013e3182a501e3

[17] Kotagal M , Symons RG , Hirsch IB , et al. Perioperative hyperglycemia and risk of adverse events among patients with and without diabetes. Ann Surg 2015;261:97–103. 25133932 10.1097/SLA.0000000000000688 PMC4208939

[18] Jackson RS , Amdur RL , White JC , Macsata RA . Hyperglycemia is associated with increased risk of morbidity and mortality after colectomy for cancer. J Am Coll Surg 2012;214:68–80. 22079879 10.1016/j.jamcollsurg.2011.09.016

[19] NICE‐SUGAR Study Investigators , Finfer S , Liu B , et al. Hypoglycemia and risk of death in critically ill patients. N Engl J Med. 2012;367:1108–18. 22992074 10.1056/NEJMoa1204942

[20] NICE‐SUGAR Study Investigators , Finfer S , Chittock DR , et al. Intensive versus conventional glucose control in critically ill patients. N Engl J Med. 2009;360:1283–97 19318384 10.1056/NEJMoa0810625

[21] Martin ET , Kaye KS , Knott C , et al. Diabetes and risk of surgical site infection: a systematic review and meta‐analysis. Infect Control Hosp Epidemiol 2016;37:88–99. 26503187 10.1017/ice.2015.249 PMC4914132

[22] Kao LS , Meeks D , Moyer VA , Lally KP . Peri‐operative glycaemic control regimens for preventing surgical site infections in adults. Cochrane Database Syst Rev 2009;8:CD006806. 10.1002/14651858.CD006806.pub2 PMC2893384 19588404

[23] de Vries FE , Gans SL , Solomkin JS , et al. Meta‐analysis of lower perioperative blood glucose target levels for reduction of surgical‐site infection. Br J Surg 2017;104:e95–e105. 27901264 10.1002/bjs.10424

[24] van den Berghe G , Wouters P , Weekers F , et al. Intensive insulin therapy in critically ill patients. N Engl J Med 2001;345:1359–67. 11794168 10.1056/NEJMoa011300

[25] Treggiari MM , Karir V , Yanez ND , Weiss NS , Daniel S , Deem SA . Intensive insulin therapy and mortality in critically ill patients. Crit Care 2008;12:R29. 18312617 10.1186/cc6807 PMC2374630

[26] Kansagara D , Fu R , Freeman M , Wolf F , Helfand M . Intensive insulin therapy in hospitalized patients: a systematic review. Ann Intern Med 2011;154:268–82. 21320942 10.7326/0003-4819-154-4-201102150-00008

[27] Van den Berghe G , Wilmer A , Hermans G , et al. Intensive insulin therapy in the medical ICU. N Engl J Med 2006;354:449–61. 16452557 10.1056/NEJMoa052521

[28] Yamada T , Shojima N , Noma H , Yamauchi T , Kadowaki T . Glycemic control, mortality, and hypoglycemia in critically ill patients: a systematic review and network meta‐analysis of randomized controlled trials. Intensive Care Med 2017;43:1–15. 27637719 10.1007/s00134-016-4523-0

[29] Wiener RS , Wiener DC , Larson RJ . Benefits and risks of tight glucose control in critically ill adults: a meta‐analysis. JAMA 2008;300:933–44. 18728267 10.1001/jama.300.8.933

[30] Dellinger RP , Levy MM , Rhodes A , et al. Surviving sepsis campaign: international guidelines for management of severe sepsis and septic shock: 2012. Crit Care Med 2013;41:580–637. 23353941 10.1097/CCM.0b013e31827e83af

[31] Tsuchida T , Takesue Y , Ichiki K , et al. Influence of peri‐operative hypothermia on surgical site infection in prolonged gastroenterological surgery. Surg Infect (Larchmt) 2016;17:570–6. 27027205 10.1089/sur.2015.182

[32] Centers for Disease Control and Prevention . CDC/NHSN surveillance definitions for surgical site infections. Published 2014 Accessed August 10, 2014. Available from http://www.cdc.gov/nhsn/pdfs/pscmanual/17pscnosinfdef_current.pdf.

[33] Okabayashi T , Shima Y , Sumiyoshi T , et al. Intensive versus intermediate glucose control in surgical intensive care unit patients. Diabetes Care 2014;37:1516–24. 24623024 10.2337/dc13-1771

[34] Van den Berghe G , Wilmer A , Milants I , et al. Intensive insulin therapy in mixed medical/surgical intensive care units: benefit versus harm. Diabetes 2006;55:3151–9. 17065355 10.2337/db06-0855

[35] Jacobi J , Bircher N , Krinsley J , et al. Guidelines for the use of an insulin infusion for the management of hyperglycemia in critically ill patients. Crit Care Med 2012;40:3251–76. 23164767 10.1097/CCM.0b013e3182653269

[36] Furnary AP , Gao G , Grunkemeier GL , et al. Continuous insulin infusion reduces mortality in patients with diabetes undergoing coronary artery bypass grafting. J Thorac Cardiovasc Surg 2003;125:1007–21. 12771873 10.1067/mtc.2003.181

[37] Kynoch K . Implementation of a glucose management protocol to prevent hypo‐ and hyperglycaemia in critically ill patients. Int J Evid Based Healthc 2008;6:468–75. 21631838 10.1111/j.1744-1609.2008.00116.x

[38] Boord JB , Sharifi M , Greevy RA , et al. Computer‐based insulin infusion protocol improves glycemia control over manual protocol. J Am Med Inform Assoc 2007;14:278–87. 17329722 10.1197/jamia.M2292 PMC2244871

[39] Saur NM , Kongable GL , Holewinski S , O'Brien K , Nasraway SA Jr . Software‐guided insulin dosing: tight glycemic control and decreased glycemic derangements in critically ill patients. Mayo Clin Proc 2013;88:920–9. 24001484 10.1016/j.mayocp.2013.07.003

